# Comment on “Odontogenic Tumors: A Challenge for Clinical Diagnosis and an Opportunity for AI Innovation”

**DOI:** 10.30476/dentjods.2024.103266.2437

**Published:** 2025-03-01

**Authors:** Hinpetch Daungsupawong, Viroj Wiwanitkit

**Affiliations:** 1 Private Academic Consultant, Phonhong, Lao People's Democratic Republic, Laos, Indian; 2 Dept. of Research Analytics, Saveetha Dental College and Hospitals, Saveetha Institute of Medical and Technical Sciences Saveetha University, Saveetha, Indian

## Dear Editor

“Odontogenic Tumors: A Challenge for Clinical Diagnosis and an Opportunity for AI Innovation. [ 1 ]” is informative and we would like to comment on this article. In this article, the authors discussed the challenges associated with clinical diagnosis of odontogenic tumors and highlighted the potential for artificial intelligence (AI) innovation in this field. While the authors did well in emphasizing the complexities of diagnosing these tumors and the need for advanced technology, they could have provided more details on the specific types of odontogenic tumors that pose the greatest challenges for diagnosis. Additionally, a more thorough exploration of the current limitations in diagnosing these tumors would have provided a more comprehensive understanding of the issue.

Moving forward, future research should focus on developing AI algorithms that can accurately differentiate between different types of odontogenic tumors based on their unique characteristics. By incorporating advanced imaging techniques and machine learning algorithms, researchers can improve the accuracy and efficiency of diagnostic processes. Moreover, studies should also investigate the potential benefits of using AI in treatment planning and monitoring of odontogenic tumors to enhance patient outcomes.

In terms of recommendations, it is essential for healthcare professionals to receive adequate training and education on the latest advancements in AI technology for diagnosing odontogenic tumors. Collaboration between dental professionals and AI experts is crucial in developing innovative solutions that can streamline the diagnostic process and improve patient care. Additionally, further research should be conducted to evaluate the cost-effectiveness and feasibility of implementing AI- based diagnostic tools in clinical practice.

Combining AI with other cutting-edge technologies, such as genomics and proteomics, could be a different and possibly more successful method of examining odontogenic malignancies. By integrating these several fields of study, scientists can develop more accurate diagnostic and therapeutic approaches by gaining a deeper comprehension of the molecular pathways driving odontogenic malignancies. In the future, this multidisciplinary approach has the potential to drastically enhance patient outcomes and transform the dental industry.
